# A method to culture human alveolar rhabdomyosarcoma cell lines as rhabdospheres demonstrates an enrichment in stemness and Notch signaling

**DOI:** 10.1242/bio.050211

**Published:** 2021-02-09

**Authors:** Katherine K. Slemmons, Michael D. Deel, Yi-Tzu Lin, Kristianne M. Oristian, Nina Kuprasertkul, Katia C. Genadry, Po-Han Chen, Jen-Tsan Ashley Chi, Corinne M. Linardic

**Affiliations:** 1 Departments of Pharmacology and Cancer Biology, Duke University School of Medicine, Durham, North Carolina; 2 Pediatrics, Duke University School of Medicine, Durham, North Carolina; 3 Duke University, Durham, North Carolina; 4 Molecular Genetics and Microbiology, Duke University School of Medicine, Durham, North Carolina

**Keywords:** Fusion-positive rhabdomyosarcoma, Spheres, Stemness, SOX2, POU5F1/OCT4, NANOG, NOTCH

## Abstract

The development of three-dimensional cell culture techniques has allowed cancer researchers to study the stemness properties of cancer cells in *in vitro* culture. However, a method to grow PAX3-FOXO1 fusion-positive rhabdomyosarcoma (FP-RMS), an aggressive soft tissue sarcoma of childhood, has to date not been reported, hampering efforts to identify the dysregulated signaling pathways that underlie FP-RMS stemness. Here, we first examine the expression of canonical stem cell markers in human RMS tumors and cell lines. We then describe a method to grow FP-RMS cell lines as rhabdospheres and demonstrate that these spheres are enriched in expression of canonical stemness factors as well as Notch signaling components. Specifically, FP-RMS rhabdospheres have increased expression of *SOX2*, *POU5F1 (OCT4),* and *NANOG,* and several receptors and transcriptional regulators in the Notch signaling pathway. FP-RMS rhabdospheres also exhibit functional stemness characteristics including multipotency, increased tumorigenicity *in vivo*, and chemoresistance. This method provides a novel practical tool to support research into FP-RMS stemness and chemoresistance signaling mechanisms.

## Abstract (precis)

**Summary:** Here we report on a method to culture human PAX3-FOXO1 fusion-positive rhabdomyosarcoma cells in three dimensions, and use these rhabdospheres as a novel tool to study their stemness and chemoresistance signaling mechanisms.

## INTRODUCTION

Relapsed and refractory disease remain dire problems in the field of clinical oncology. Cancer stem cells (CSC), or cancer cells exhibiting stemness properties and sometimes called tumor-initiating cells, have emerged as the subset of chemoresistant cells thought to propagate tumor resistance and recurrence (Moitra, 2015; Morrison et al., 2011)]. Understanding the biology of CSCs is key to developing novel agents to target them; having model systems that support the growth and study of CSCs is therefore critical.

Methods to culture cancer cells as non-adherent spheres, e.g. breast cancer cells as mammospheres (Lee et al., 2007) and brain cancer cells as neurospheres (Lee et al., 2006), or other three-dimensional (3D) structures has revolutionized the field of CSC biology. Compared to the traditional method of culturing cells as adherent monolayers, culturing cells in 3D more closely recapitulates the cell–cell contact and physical forces that occur in human tumors *in situ* (Chen et al., 2012). These modified cell culture methods not only enable the study of cancer stemness, but may also provide a practical experimental method intermediate between adherent cell culture and *in vivo* xenografts (Hoarau-Vechot et al., 2018). However, sphere culture systems have yet to be developed for all human tumor types, including many sarcomas.

Sarcomas are a heterogeneous group of mesenchymal tumors that occur throughout the human lifespan. Successful generation of several sarcoma sphere systems [osteosarcoma (Basu-Roy et al., 2012), leiomyosarcoma (Sette et al., 2012), and a range of musculoskeletal sarcomas (Salerno et al., 2013)] demonstrate that they are achievable, and that their implementation has allowed researchers to unravel novel molecular mechanisms that drive cancer cell stemness in sarcomas.

Rhabdomyosarcoma (RMS) is the most common soft tissue sarcoma in children and adolescents. RMS is comprised of two main histologic subtypes, known as embryonal RMS (ERMS) and alveolar RMS (ARMS) (Ognjanovic et al., 2009). As genomic profiling of RMS has matured, the nomenclature for RMS has shifted towards molecular rather than histologic classification. Thus, ERMS tumors are termed fusion-negative (FN) RMS, referring to the lack of signature fusion oncogenes, while ARMS are termed fusion-positive (FP) RMS, referring to the presence of signature fusion oncogenes. This nomenclature will be used here.

By culturing FN-RMS cells as rhabdospheres, Walter and colleagues identified the transmembrane glycoprotein prominin 1 (PROM1, also known as CD133 antigen) as a hallmark of FN-RMS stemness (Walter et al., 2011). Subsequently, several developmental pathways including Notch, Hedgehog, and Hippo have been studied in FN-RMS rhabdospheres (Ignatius et al., 2017; Satheesha et al., 2016; Slemmons et al., 2017), demonstrating the utility of this 3D system for studying the role of dysregulated signaling in CSC stemness in FN-RMS. However, less is known about the signaling that controls stemness in FP-RMS tumorigenesis. This is important because FP-RMS, which is most often driven by the signature *PAX3-FOXO1* fusion gene, is the more aggressive variant, with a 5 year survival rate of less than 50% overall (Ognjanovic et al., 2009) and less than 17% for relapsed cases (Pappo et al., 1999). Most patients with PAX3-FOXO1-RMS will initially respond to therapy but typically become chemo-refractory or relapse (Ognjanovic et al., 2009; Missiaglia et al., 2012), underscoring the need to better understand its CSC biology. Previously, a method was reported to grow PAX7-FOXO1-positive CW9019 cells (Almazán-Moga et al., 2017). Here, we have developed a method for culturing PAX3-FOXO1-positive-RMS cells as rhabdospheres (which we abbreviate as FP-RMS), and in these spheres we observe enrichment of canonical stemness markers and Notch signaling. Moreover, culturing these FP-RMS cell lines as spheres increases their potential to differentiate into multiple cell lineages, their tumorigenic potential as xenografts in immune deficient mice, and their resistance to a standard RMS-directed chemotherapeutic agent vincristine.

## RESULTS

### FP-RMS tumors express canonical stem cell genes

**Fig. 1.**
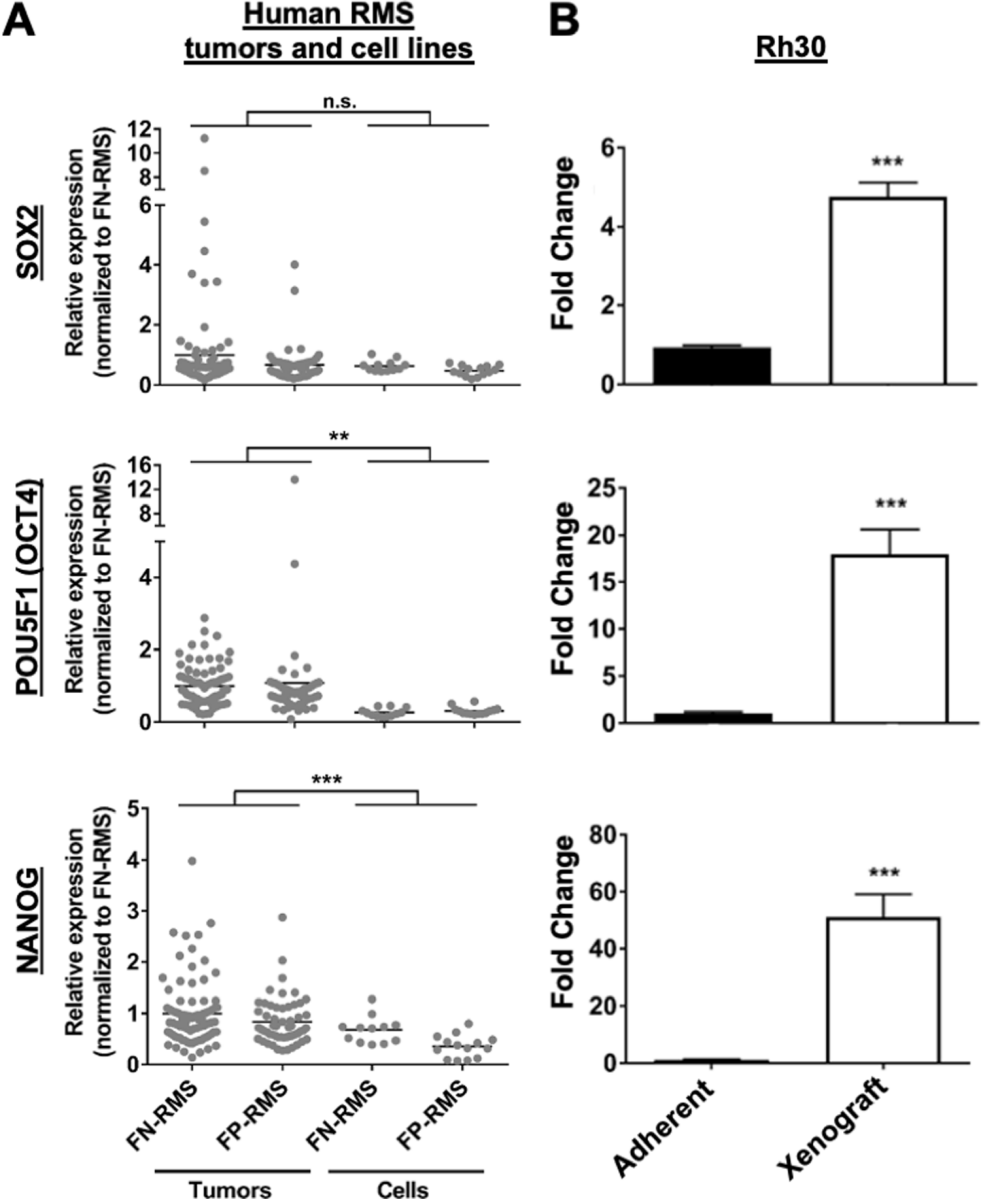
**Human FP-RMS tumors and xenografts express *SOX2*, *POU5F1* (*OCT4*), and *NANOG.*** (A) FN-RMS and FP-RMS human tumors express similar levels of *SOX2*, *POU5F1*, and *NANOG*. Values are much lower for FN-RMS and FP-RMS cells grown as cell monolayers. Microarray data was obtained from the Oncogenomics database (https://pob.abcc.ncifcrf.gov/cgi-bin/JK). *N*=84 FN-RMS tumors, *N*=55 FP-RMS tumors, *N*=12 FN-RMS cell lines, *N*=14 FP-RMS cell lines. (B) qRT-PCR demonstrates a significant increase in *SOX2*, *POU5F1*, and *NANOG* levels in Rh30 orthotopic xenografts as compared to Rh30 adherent cell cultures. *N*=3 per group. *P<0.05; **P<0.01; ***P<0.001.

Little information exists regarding stemness genes and properties in FP-RMS. Therefore, we first queried human FP-RMS tumors for mRNA expression of several canonical stem cell genes, *SOX2* (*sex determining region Y-Box 2*)*, POU domain, class 5, transcription factor 1* (*POU5F1*; also known as *OCT4*) and *Nanog homeobox* (*NANOG*). Although these genes were previously shown to be expressed in FN-RMS cell lines and tumors and serve as markers of FN-RMS cell stemness (Walter et al., 2011; Satheesha et al., 2016; Slemmons et al., 2017), the relative expression of these genes in FP-RMS was not known. We found that all three genes were expressed in human FP-RMS tumors at levels similar to the FN-RMS tumors (Fig. 1A), with the increases in *POU5F1* and *NANOG* in tumors compared to cell lines meeting statistical significance. We next evaluated expression of these genes in Rh30 cells grown *in vitro* as a monolayer versus Rh30 cells grown as a monolayer and then injected *in vivo* as orthotopic xenografts. Compared to *in vitro* culturing of cells, the xenografts had 4–50 fold increase in *SOX2, POU5F1,* and *NANOG* gene expression (Fig. 1B)*.* These data suggest that when studying CSC biology, growth as a monolayer does not accurately represent the gene expression that would be seen *in vivo*. However, because xenograft studies are costly and use animals, we sought to develop an intermediate method of studying FP-RMS cancer cell stemness *in vitro*.

### FP-RMS spheres are enriched in stemness markers and Notch signaling components

**Fig. 2.**
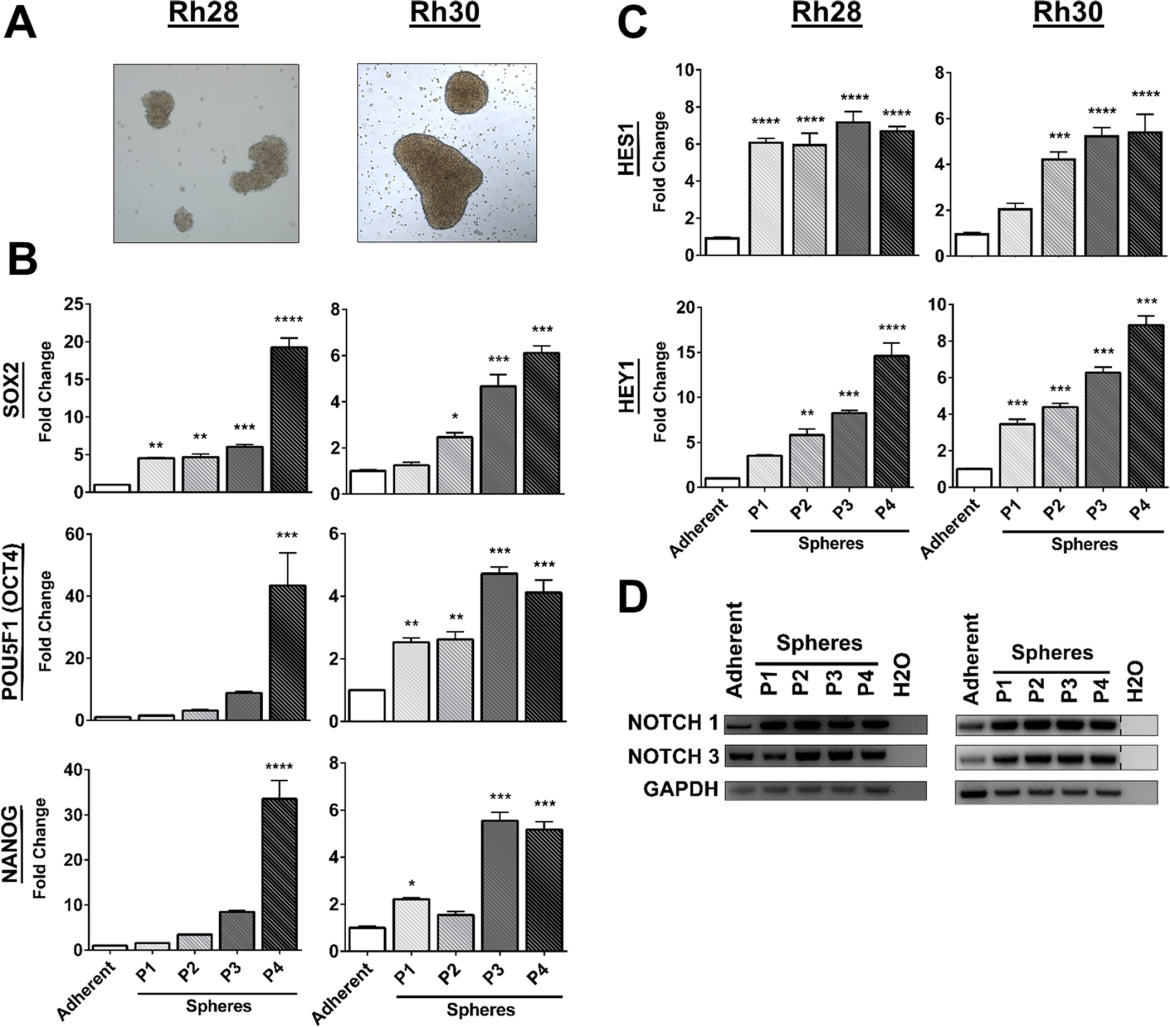
**FP-RMS rhabdospheres are enriched in stemness marker expression and Notch signaling.** (A) Representative images of Rh28 (left) and Rh30 (right) spheres. (B) Expression of stem cell markers *SOX2, POU5F1,* and *NANOG* increases in FP-RMS rhabdospheres over four passages (compared to adherent cells) as assayed by qRT-PCR. (C) Notch target genes *HES1* and *HEY1* are also increased. (D) Notch receptors *NOTCH1* and *NOTCH3* are increased as shown by RT-PCR. GAPDH used as loading control. Lanes are from the same RT-PCR experiment but have been rearranged into this order. *P*= passage number. *, *P*<0.05; **, *P*<0.01; ***, *P*<0.001; and ****, *P*<0.0001.

We first attempted to culture two PAX3-FOXO1-positive patient-derived FP-RMS cell lines (Rh30 and Rh28 cells) as spheres using a previously published FN-RMS sphere protocol (Walter et al., 2011). However, these conditions did not support rhabdosphere formation of FP-RMS cells. Recently, PAX3-FOXO1 has been shown to ‘trap’ FP-RMS cells in a myoblastic state (Gryder et al., 2017), in part through the upregulation of insulin-like growth factor 1 receptor pathways (Ayalon et al., 2001; Aslam et al., 2013). Consistent with this observation, we noted that human skeletal muscle myoblast culture medium is formulated with insulin and other insulin-like growth factors, and we therefore modified the FN-RMS sphere media by increasing the concentration of insulin and growth factors bFGF and EGF; this modified media allowed Rh30 cells to form spheres in ultra-low attachment plates (Fig. 2A, right). For the Rh28 cells, which grow slower than the Rh30 cells, increasing the B27 supplement to 2X was required for sphere formation (Fig. 2A, left).

We next investigated whether culturing FP-RMS cells as rhabdospheres enriched in stemness markers, as assessed by qRT-PCR. Compared to cells grown as monolayers, serial passaging of FP-RMS spheres led to upregulation of *SOX2*, *POU5F1*, and *NANOG* in both Rh28 and Rh30 cells (Fig. 2B). The magnitude of gene expression increases was higher in Rh28 compared to Rh30 cells (20–40-fold increase versus 4–6-fold increase at passage 4). Interestingly, Prominin 1, or CD133 (*PROM1*), which is a hallmark of FN-RMS cell stemness, was not upregulated in FP-RMS spheres.

The Notch pathway is a developmental signaling network that regulates stem cell identity and proliferation in many tissues including skeletal muscle (Bray, 2016). In FN-RMS, the role of Notch has been better characterized and shown to play a role in regulating stemness (Ignatius et al., 2017; Slemmons et al., 2017). However, the role of Notch in FP-RMS and FP-RMS stemness is not well understood. We analyzed expression of two Notch signaling readout genes *hes family bHLH transcription factor 1* (*HES1*) and *hairy/enhancer-of-split related with YRPW motif 1* (*HEY1*)*,* and two Notch receptors, *NOTCH1* and *NOTCH3.* We found that all four Notch pathway genes were increased in the spheres (Fig. 2C,D), with the magnitude of increase again more pronounced in the Rh28 cells. These data suggest that Notch may not only be important for FN-RMS, but also for FP-RMS, and that sphere culture systems may be a useful tool to study Notch signaling in FP-RMS.

### FP-RMS spheres are multipotent

To further characterize the stem cell characteristics of the FP-RMS rhabdospheres, we evaluated their multipotency, or ability to differentiate into multiple lineages. Since RMS is thought to originate from a mesenchymal precursor (Naini et al., 2008; Abraham et al., 2014), enriching for stem cell characteristics by culturing as spheres should promote the ability of FP-RMS cells to differentiate along various mesenchymal lineages (myogenic, adipogenic, neurogenic and osteogenic lineages). Previous studies have shown that native FP-RMS cells grown as an adherent monolayer do not exhibit stem cell qualities and are not readily able to differentiate (Lee et al., 2011).

**Fig. 3.**
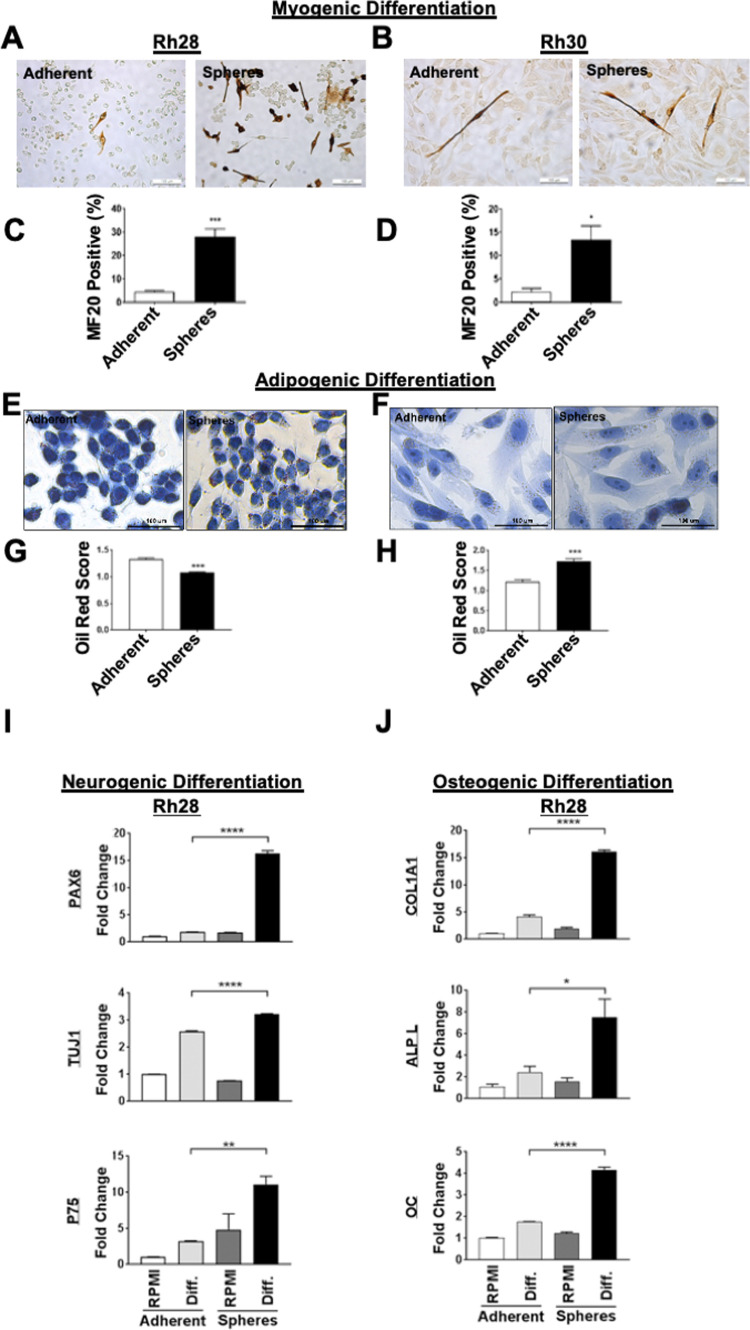
**FP-RMS spheres are multipotent.** Cells cultured in adherent or spheres conditions were grown in myogenic differentiation media for 5 days then stained for MF20 expression. (A,B) Representative images and (C,D) quantitation of MF20 staining in Rh28 and Rh30 cells. After adipogenic differentiation, cells were stained with Oil Red O. (E,F) Representative images and (G,H) scoring of Oil Red O staining in Rh28 and Rh30 cells. (I,J) qRT-PCR analysis of neurogenic and osteogenic differentiation genes in Rh28 cells from adherent versus sphere culture conditions, after neurogenic and osteogenic differentiation, respectively. *P<0.05; **P<0.01; ***P<0.001; and ****P<0.0001.

We found that Rh28 and Rh30 cells cultured as spheres prior to re-plating in myogenic differentiation media demonstrated a greater ability to differentiate along the myogenic lineage than cells that had been cultured as an adherent monolayer (Fig. 3A,B). Quantitation of MF20 staining, which identifies myosin heavy chain, showed that only 2–4% of the adherent cell population was MF20 positive, while 13–26% of cells that had been cultured as spheres were able to differentiate (Fig. 3C,D), demonstrating the increased plasticity of cells grown as spheres.

Results for differentiation along the adipogenic lineage were mixed. After 11 days in adipogenic differentiation media, lipid droplets were identified using Oil Red O staining (Fig. 3E,F) and the relative amount of lipid droplets was scored and quantified (Fig. 3G,H). The Rh30 cells that had been cultured as spheres displayed a higher degree of adipogenic differentiation, as shown by a higher average Oil Red O score, than the cells that had been cultured as an adherent monolayer. However, this finding was not observed in the Rh28 cells, for which a slightly higher number of lipid droplets was observed in the cells that had been grown adherently. Therefore, to further investigate the pluripotency of the Rh28 spheres, we performed two additional differentiation assays along the mesenchymal lineage.

Rh28 cells that had been grown either as an adherent monolayer or as spheres were cultured in neurogenic or osteogenic differentiation conditions. Using qRT-PCR, mRNA expression for markers of neurogenic [*paired box 6* (*PAX6*)*, TUJ1* (*TUBB3, tubulin, beta 3 class III*) *and P75 nerve growth factor receptor*, *NGFR*] and osteogenic [*collagen type I alpha 1 chain* (*COL1A1*)*, alkaline phosphatase* (*ALPL*) *and OC (bone gamma-carboxyglutamate protein*, *BGLAP*] differentiation were analyzed. Although a small percentage of cells that were grown adherently differentiated in the neurogenic and osteogenic differentiation media, a significantly higher expression of each of these genes was observed in the cells that had been cultured as spheres (Fig. 3I,J). These data suggest that culturing FP-RMS cells as spheres permits plasticity, allowing for differentiation down different mesenchymal lineages.

We considered the possibility that the different culture conditions induced, rather than just permitted, stemness and plasticity. To test this, we cultured normal human skeletal muscle myoblasts (HSMM) and Rh30 cells in sphere media in ultra-low attachment plates or adherent plates, and compared them to HSMMs and Rh30s grown in standard growth media on adherent plates. While the Rh30 cells were able to be passaged in all conditions, and formed spheres when grown in sphere media on ultra-low attachment plates, HSMMs did not survive when grown in sphere media in either ultra-low attachment plates or adherent plates (Fig. S1). These data suggest that cells that lack an inherent plasticity or stemness are not induced to reprogram to a stem-like state by growth in these culture conditions.

### FP-RMS spheres demonstrate increased tumorigenicity *in vivo*

**Fig. 4.**
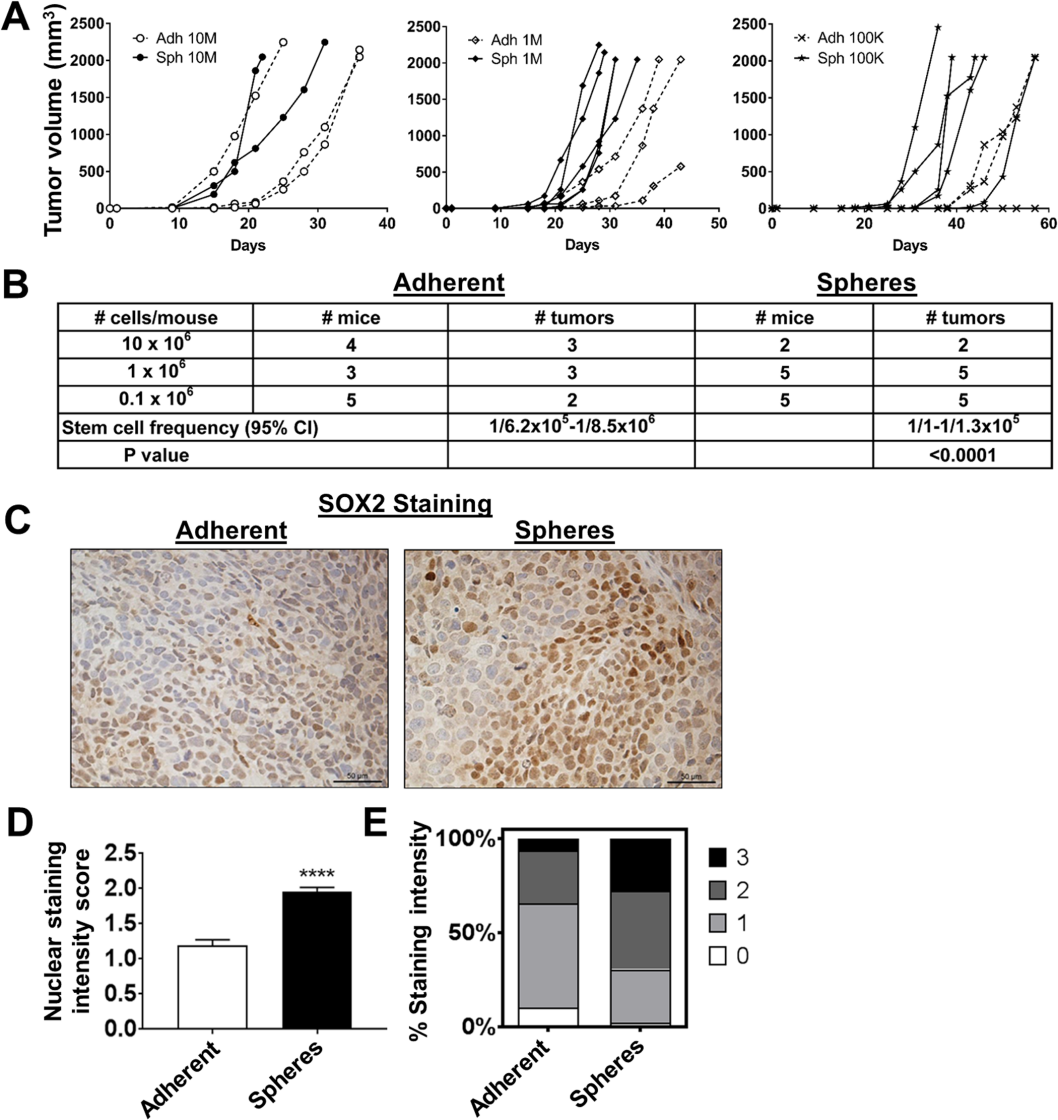
**FP-RMS spheres exhibit increased tumorigenicity *in vivo.*** (A) Tumor volume over time of mice injected subcutaneously with 10×10^6^ (left), 1 x10^6^ (middle), or 1×10^5^ (right) Rh30 adherent or sphere cells. (B) The number of tumors per group is shown, with spheres having a higher penetrance. ELDA software was used to calculate the stem cell frequency. (C) Representative images of SOX2 IHC in the adherent and sphere tumors, which scored 1 and 2, respectively on IHC scoring. (D) Quantitation of SOX2 IHC staining. (E) Distribution of nuclear staining scores between adherent and sphere tumors. 0, negative (no brown staining); 1, weak staining; 2, moderate staining; 3, strong staining. ****P<0.0001.

To evaluate the impact of culturing human FP-ARMS cells as spheres on *in vivo* tumorigenesis, we performed xenograft assays whereby varying doses of Rh30 cells (grown adherently or as spheres) were injected subcutaneously into the flanks SCID/*beige* mice. At the highest cell dose (10×10^6^), cells from both adherent and sphere conditions formed tumors with similar penetrance and kinetics (Fig. 4A, left). At the intermediate cell dose (1×10^6^), while all the mice developed tumors, the adherent group grew slower (Fig. 4A, middle). At the lowest dose (1×10^5^), all mice injected from cells that had been cultured as spheres formed tumors while only 2/5 mice from the adherent group developed tumors (Fig. 4A, right). Statistical analysis of tumor formation from the sphere group demonstrated a significantly higher estimated stem cell frequency compared to the adherent group (Fig. 4B). These data demonstrate that stemness is functionally enriched through this *in vitro* method of culturing FP-RMS cells as 3D rhabdospheres.

To assess whether the *in vitro* stemness enrichment was maintained *in vivo* after tumor formation, we evaluated the harvested tumors for SOX2 protein expression using IHC. Compared to the tumors from the adherent group, the tumors from the sphere group showed increased nuclear SOX2 protein staining **(**Fig. 4C–E). Using qRT-PCR, we analyzed mRNA expression of stem cell markers *SOX2, POU5F1,* and *NANOG* within the tumors. Interestingly, the tumors arising from the sphere group were not different from tumors arising from the adherent group in mRNA expression of any of the stem cell genes (Fig. S2A–C), nor in the Notch target genes *HES1* and *HEY1* (Fig. S2D,E). We do not know the reason for this, but posit that rhabdosphere conditions promote tumor development through an enrichment in stemness, and once tumors are established, there is no longer a need for active transcription of these stem cell genes. In summary, culturing of FP-RMS cells as spheres increases their tumorigenicity and expression of SOX2 at the protein, but not transcript, level.

### FP-RMS spheres exhibit resistance to chemotherapy

**Fig. 5.**
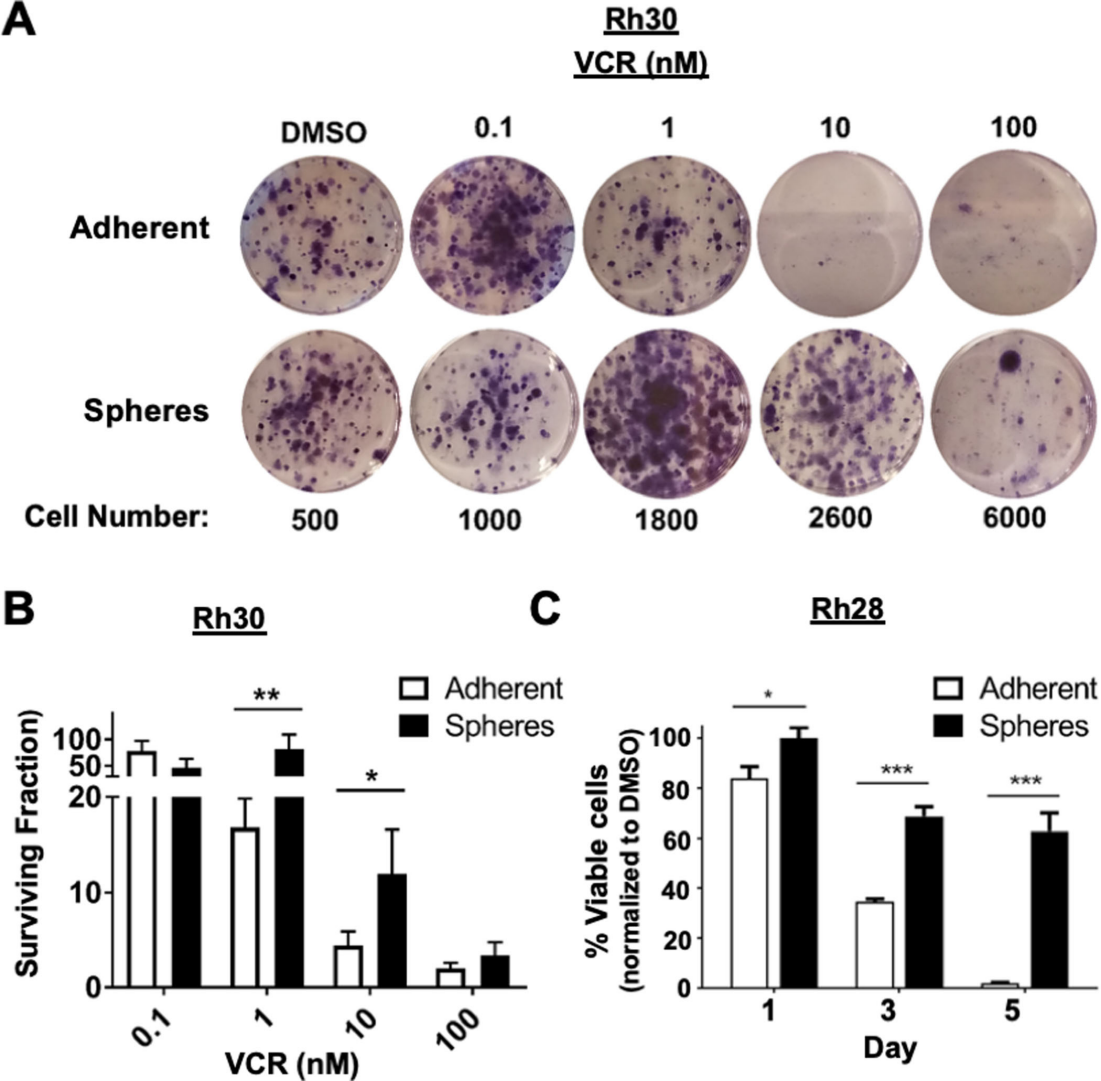
**FP-RMS spheres have increased chemoresistance.** (A) Representative images of Crystal violet staining of Rh30 cells after treatment with VCR. The cell number plated is shown below as ‘Cell Number’. (B) Quantitation of Crystal violet staining normalized to DMSO control. (C) Percent of viable Rh28 cells after VCR treatment over 5 days. *P<0.05; **P<0.01; ***P<0.001.

Cancer stem cells are thought to be the chemoresistant subpopulation within a tumor. In other cancers including FN-RMS, sphere culturing *in vitro* results in increased chemoresistance (Walter et al., 2011; Li et al., 2008; Xia et al., 2014). In line with this, we evaluated whether FP-RMS spheres exhibit resistance to vincristine (VCR), an anti-microtubule agent that is part of the backbone of RMS chemotherapy (Arndt et al., 2009), compared to cells grown as a monolayer. Rh30 adherent cells versus spheres were treated with DMSO or increasing concentrations of VCR (0.1–100 nM), plated into a colony formation assay, and stained with Crystal violet. While the adherent cells were sensitive to VCR even at low concentrations, as demonstrated by fewer colonies, the spheres were more resistant to VCR as demonstrated by the increased number of colonies even at very high concentrations of VCR (Fig. 5A). Quantitation of the area of Crystal violet staining similarly demonstrated that the spheres were more resistant to chemotherapy than the monolayers (Fig. 5B).

Quantification of the chemoresistance of Rh28 spheres was more challenging. Rh28 cells do not grow well under sparsely plated conditions, which is the condition that is the basis of the colony formation assay. However, at the end of the colony formation assay, while all the adherent cells died at 10 nM and 100 nM VCR, we observed the emergence of a few resistant clones in the Rh28 spheres (dark cells representing the dead, floating cells; bright cells representing surviving clones) (Fig. S3). Images were taken using light microscopy prior to Crystal violet staining. Since there were only a few colonies in the sphere conditions, and no live cells remained in the adherent conditions, it was difficult to discern Crystal violet colonies above background staining. To further investigate the chemoresistance of Rh28 spheres, we analyzed cell viability over time by Trypan blue cell counting after VCR treatment (Fig. 5C). While the cell viability of the adherent cells decreased over 5 days following VCR treatment, more than 60% of the Rh28 cells cultured as spheres remained viable at day 5. Overall, these data suggest the FP-RMS spheres exhibit chemoresistance and therefore would be a useful system to study chemoresistance and to test novel agents that target RMS cancer stem cells. Since stemness is associated with slower proliferation (Kleffel and Schatton, 2013), it is possible that FP-RMS cells grown as spheres were chemoresistant due in part to this, but a recent study in leukemia showed that VCR induces toxicity even in interphase (Kothari et al., 2016), suggesting that the chemoresistance demonstrated by stem cells is multifactorial. Further studies are needed to parse out the mechanisms of chemoresistance in FP-RMS cells grown as spheres.

### Gene expression profile of FP-RMS spheres

**Fig. 6.**
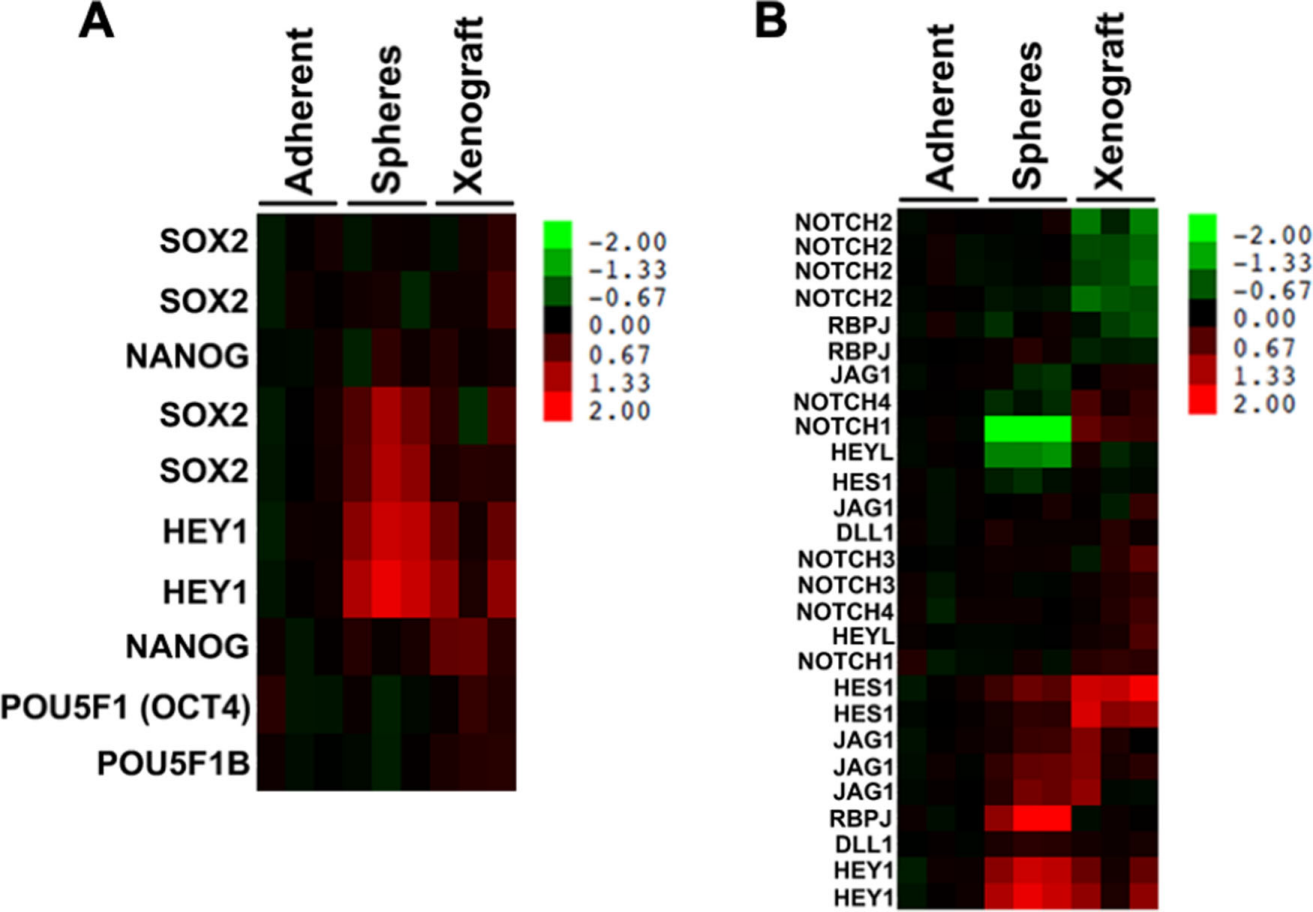
**FP-RMS spheres and xenografts show upregulation of stemness and Notch genes.** Microarray analysis of Rh30 cells grown in adherent, sphere, and orthotopic xenograft conditions reveals an upregulation of (A) stem cell genes and (B) Notch signaling in both the spheres and xenografts as compared to the adherent cells.

To gain further insight into the consequences of culturing FP-RMS cells as rhabdospheres versus other systems, we performed microarray analysis of RNA isolated from Rh30 cells grown as adherent monolayers, rhabdospheres, or orthotopic xenografts (established from adherent cultures) in biological triplicate. When compared to FP-RMS cells cultured adherently, FP-RMS cells cultured *in vitro* as spheres or grown *in vivo* as xenografts demonstrate enrichment in stem cell genes (*SOX2*, *POU5F1* and *NANOG*) (Fig. 6A). FP-RMS cells cultured *in vitro* as spheres or grown *in vivo* as xenografts also demonstrated enrichment in Notch signaling components [*HEY1*, *HES1*, jagged 1 (*JAG1*) and recombination signal binding protein for immunoglobulin kappa J region (*RBPJ*)], as well as a modest increase in *NOTCH3* and *NOTCH4* (Fig. 6B). These data are similar to our qRT-PCR data and further suggest that the FP-RMS rhabdosphere system can recapitulate the stem cell and Notch gene expression profiles of FP-RMS tumors.

## DISCUSSION

Here we report a method for culturing human PAX3-FOXO1-positive (FP-RMS cells) as 3D rhabdospheres, and our use of this method to investigate the properties of FP-RMS when grown as spheres. We find that FP-RMS cells grown as spheres compared to monolayers exhibit higher expression of canonical stemness markers and Notch signaling components, and functionally demonstrate increased multipotency, enhanced ability to grow as xenografts, and increased chemoresistance. This is the first report describing the culture of PAX3-FOXO1-positive RMS cells as spheres along with enrichment of a CSC population.

The implications for this work are both technical and conceptual. Technically, this FP-RMS sphere protocol provides a tool with which to interrogate RMS biology, allowing for the study of genes that regulate FP-RMS stemness, including differences between FN-RMS and FP-RMS. Conceptually, this work demonstrates a limitation of conventional *in vitro* adherent monolayer cell culture methods for studying genes that may regulate CSC properties. In this regard, culturing FP-RMS as spheres may be required for the study of certain biological pathways. For example, culturing of FP-RMS cells *in vitro* as 3D spheres, but not adherent monolayers, enriched for canonical stem cell and Notch components also seen expressed in FP-RMS tumors *in vivo*. While we analyzed expression of Notch pathway components, other signaling pathways such as Hedgehog (Satheesha et al., 2016; Almazán-Moga et al., 2017) might also be important in FP-RMS stemness and could be studied using this method. Culturing FP-RMS cells as spheres may also support studies of signaling cross-talk and therapeutic resistance, as was done for the study of Notch and Hippo signaling in FN-RMS stemness (Slemmons et al., 2017).

As with any new experimental system, there remain unanswered questions. However, we imagine that this model will be helpful in addressing them. For example, why was *PROM1* (CD133) found upregulated in FN-RMS (Walter et al., 2011) but not in FP-RMS? It is possible that different signaling circuits support stemness and tumor initiation in different RMS subtypes, and potentially even CD133-negative cells, as found in some carcinomas (LaBarge and Bissell, 2008; Shmelkov et al., 2008). If so, this has therapeutic implications since CSC-directed treatments (e.g. anti-CD133 biologics) designed for one subtype may not be effective in the other. Second, why do different FP-RMS cell lines require different concentrations of growth supplements to form spheres? Again, we posit that different FP-RMS cell lines rely on unique cell growth circuits and thus have differential growth factor requirements. It is also possible that the supplements included in sphere media represent *in vitro* mimics of important circuits that contribute to stemness, pluripotency and tumorigenesis *in vivo*. It will be important in future experiments to compare the biology of FP-RMS cells grown as spheres in sphere media to monolayer cells grown in sphere media, rather than conventional culture conditions, to help elucidate the relative contributions of these growth factors. Last, why do different FP-RMS cell lines show different pluripotency capacity when grown as spheres? This may highlight the phenotypic heterogeneity of FP-RMS cells, which could be mirrored in FP-tumors. While these are only hypotheses, this defined sphere model system provides a controlled system in which to interrogate these variables.

In conclusion, we report the development of a method to culture FP-RMS cells as 3D rhabdospheres. This novel method facilitates study of stemness markers and signaling, multipotency, and chemoresistance in FP-RMS.

## MATERIALS AND METHODS

### Generation of cell lines and spheres

Human FP-RMS cell lines Rh28 and Rh30 were gifts from Tim Triche (Children's Hospital of Los Angeles, CA, USA) in 2005 and both express the *PAX3-FOXO1* fusion gene (Douglass et al., 1987; Hazelton et al., 1987). Cell line authentication was performed in July 2014 and September 2016 using STR analysis (Promega GenePrint 10) conducted by the Duke University DNA Analysis Facility. A protocol to culture Rh30 cells as spheres was developed, based on modifications to the published FN-RMS sphere protocol (Walter et al., 2011). In brief, Rh30 cells were cultured in Neurobasal media supplemented with 1X B27, 80 ng/ml bFGF, 40 ng/ml EGF, and 50 µg/ml insulin. A protocol to culture Rh28 spheres was then developed in which Rh30 sphere media was instead supplemented with 2X B27. Sphere experiments were performed in either six-well, 10 cm, or 25 ml ultra-low attachment plates or flasks (Corning). Spheres were passaged (split 1:2) approximately every 48–72 h once spheres were >2 mm and/or media became acidic. Spheres were manually dissociated by pipetting at each passaging. FP-RMS cells grown as spheres were compared to FP-RMS cells grown in conventional monolayer culture conditions (RPMI 1640 plus 10% FBS). Human skeletal muscle myoblasts (HSMM) were purchased from Lonza and cultured according to manufacturer's specifications using the SkBM-2 medium and bullet kit (Lonza).

### Quantitative real time PCR and Semi-quantitative PCR

PCR was performed as described (Crose et al., 2014). Primer sets are listed in Table S1.

### Differentiation assays

To assess pluripotency, FP-RMS cells were cultured for 48 h as adherent cells versus spheres, then plated as a monolayer in order to conduct differentiation assays. Myogenic differentiation and MF20 staining were performed as described (Linardic et al., 2007). The MF20 antibody recognizes all isoforms of myosin heavy chain in differentiated skeletal muscle and was deposited to the Developmental Studies Hybridoma Bank by D.A. Fischman. Positively and negatively stained cells were counted manually with the aid of cell-counting software (ImageJ, NIH). Four images were counted per condition. Adipogenic differentiation was performed as described (Walter et al., 2011), followed by Oil Red O staining (Thermo Fisher Scientific). Cells were scored on a scale of 0–3 by a blinded scorer, four images per condition. Neurogenic differentiation was performed by plating cells on six-well plates coated with 0.01% type I collagen followed by treatment with neurogenic conditioning medium (10 nM retinoic acid and 0.5% FBS in RPMI-1640). Media was changed every other day, cells were harvested at day 21, and qRT-PCR for neurogenic differentiation makers was performed. Osteogenic differentiation was performed by plating cells on six-well plates coated with 0.01% type I collagen followed by treatment with osteogenic conditioning medium (2% FBS, 5 mM β–glycerol phosphate and 50 µg/ml vitamin C in RPMI-1640). Half of the media was replaced every other day, cells were harvested at day 24 and expression of osteogenic differentiation markers was analyzed by qRT-PCR.

### Mouse xenograft studies

For the limiting dilution assays (Fig. 4), 10×10^6^, 1×10^6^, and 1×10^5^ Rh30 cells grown as adherent cultures or as spheres were resuspended in Matrigel (BD Biosciences), implanted subcutaneously into the flanks of immunodeficient SCID/*beige* mice as previously described (Crose et al., 2014; Belyea et al., 2011). Stem cell frequency was calculated using ELDA software (Hu and Smyth, 2009). For the microarray study (Fig. 6) and PCR analysis (Fig. 1B), 1×10^6^ Rh30 cells grown as adherent cultures were resuspended in sterile PBS and injected intramuscularly into the left gastrocnemius of SCID/*beige* mice. For both studies, mice were observed twice weekly for evidence of malaise, weight loss or inability to ambulate normally, and the limbs were measured with calipers. Tumor volume was calculated as [((width×length)/2)/3]/2. Mice were sacrificed upon reaching an Institutional Animal Care and Use Committee (IACUC)-defined maximum tumor burden, weight loss exceeding 15%, or loss of ambulatory ability. Animals that did not reach experimental end points 4 months after injection were euthanized at the end of study and necropsied. Tumors were preserved in RNAlater (Qiagen) for PCR or formalin-fixed for IHC. Both studies were approved by Duke University's IACUC.

### Drug studies

Vincristine (VCR) was obtained from Selleckchem and resuspended in DMSO at 10 mM. Rh30 cells grown as an adherent monolayer versus spheres were treated with varying concentrations of VCR for 48 h and then seeded into a colony formation assay in six-well plates. After 12 days, colonies were visualized with 1% Crystal violet staining. Rh28 cells (1×10^6^ cells/plate) grown as adherent cells or as spheres were directly treated with DMSO or 1 nM VCR and cell viability was analyzed by automated cell counting and Trypan blue staining at 1, 3, and 5 days, performed in triplicate.

### Microarray

RNA samples collected from adherent cells, spheres (after 48 h in culture), and orthotopic xenografts in biological triplicates were isolated using an RNeasy kit (Qiagen) according to the manufacturer's protocol. Affymetrix U133 Plus 2.0 arrays were used and performed by the Duke University Microarray Facility according to manufacturer's instruction. CEL files of all samples were normalized by RMA Robust Multi-Array algorithm, zero-transformed against the average expression levels of the same probe sets of the Rh30 adherent control, filtered by indicated criteria, clustered with Cluster 3.0, and displayed with TreeView as previously described (Crose et al., 2014; Chen et al., 2017). All the microarray data have been submitted into Gene Expression Omnibus (GEO) with accession number GSE119716.

### Immunohistochemistry

Paraffin-embedded formalin-fixed xenograft tumor samples were sectioned and stained. SOX2 immunohistochemistry (IHC; Cell Signaling Technology #3579, 1:100) was performed as described (Ren et al., 2016). The staining was scored on a scale of 0–3 by three blinded scorers: 0, negative (no brown staining); 1, weak staining; 2, moderate staining; 3, strong staining; similar to previous work (Zhou et al., 2015). Standards for each score are reported in (Slemmons et al., 2017). Four images were scored per tumor and averaged.

### Statistical analysis

Statistical analysis was performed using GraphPad Prism (GraphPad). Unless otherwise noted, data is presented as the mean and SE. One-way ANOVA, two-way ANOVA, and unpaired *t*-test were used as appropriate. *P* values were considered significant at *P< 0.05; **P<0.01; ***P<0.001; and ****P<0.0001.

## Supplementary Material

Supplementary information
