# Supplementary material for: A method to culture human alveolar rhabdomyosarcoma cell lines as rhabdospheres demonstrates an enrichment in stemness and Notch signaling
Source: Biol Open. 2021 Feb 9;10(2):bio050211. doi: 10.1242/bio.050211 (PMC7888706; doi:10.1242/bio.050211)
Supplement: Supplementary information [file biolopen-10-050211-s1.pdf]

Supplementary Figure 1

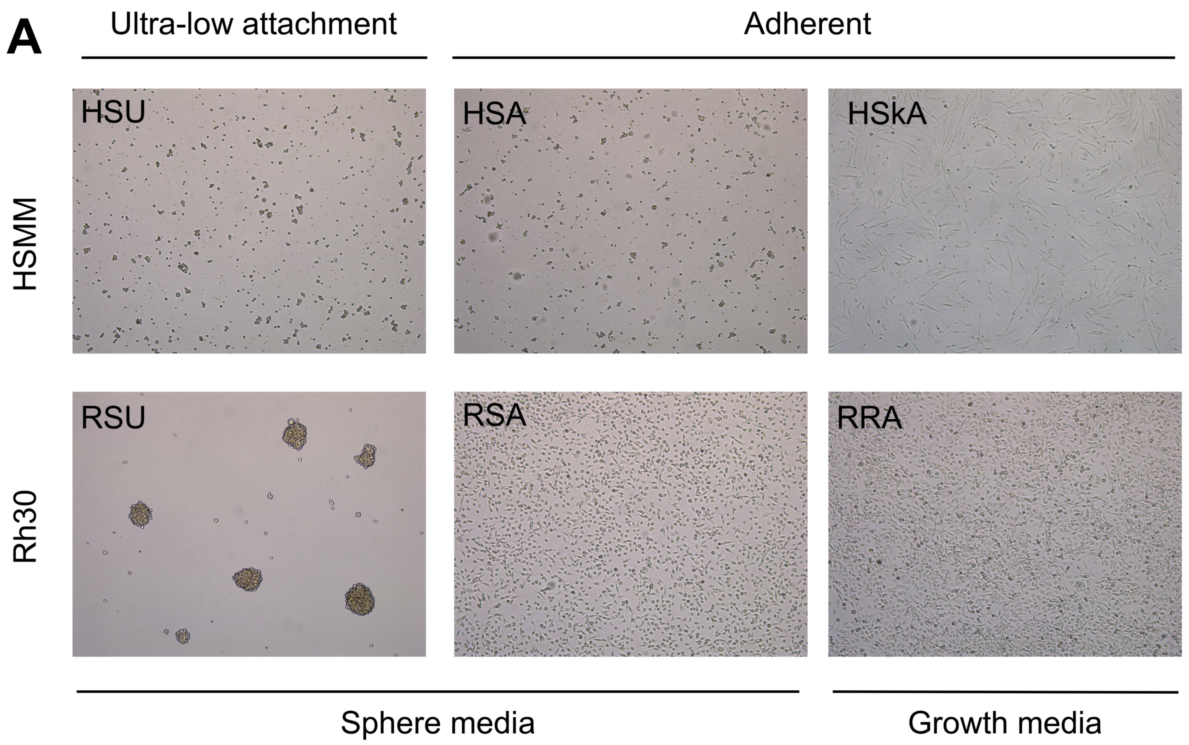

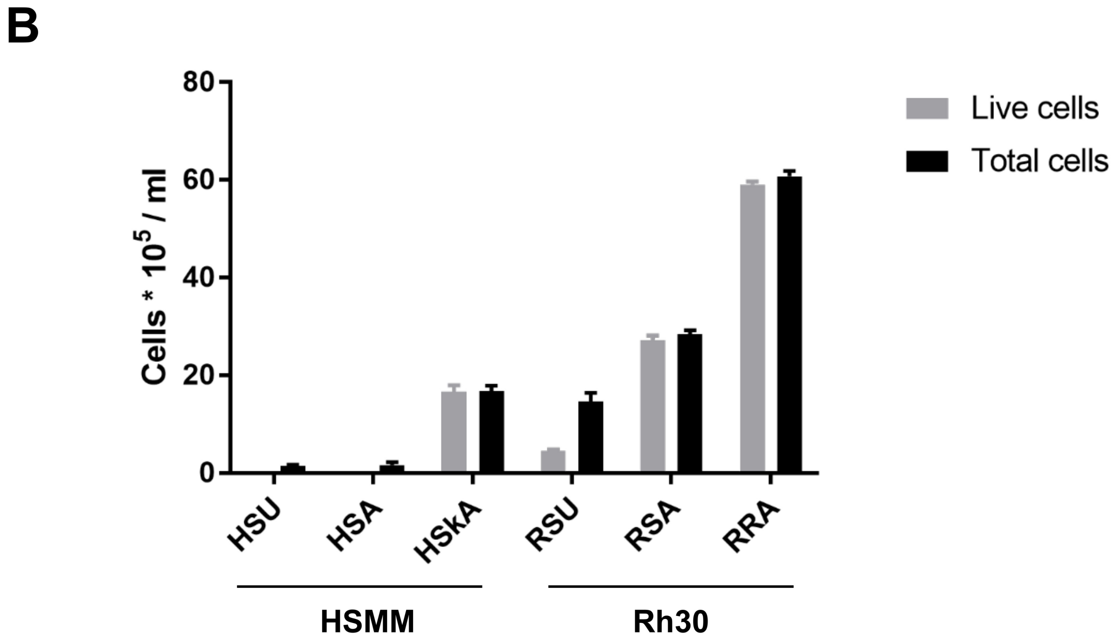

Biology Open • Supplementary information

Figure S 1. Normal human skeletal muscle myoblasts (HSMM) do not survive as spheres. (A) Representative 100x images of HSMM and Rh30 cells after culture in sphere media on ultra-low attachment plates, or sphere media on adherent plates, as compared to normal growth media on adherent plates. HSMMs were not able to be passaged in sphere media on ultra-low attachment or adherent plates, shown by the dark floating cells. **(B)** Number of live cells compared to total cells remaining after culture in sphere media on ultra-low attachment plates, or sphere media on adherent plates, as compared to normal growth media on adherent plates. Concentration is represented as 10^5 cells/ml. HSU: HSMM cells, sphere medium, ultra-low attachment plates; HSA: HSMM cells, sphere medium, adherent plates; HSkA: HSMM cells, skeletal muscle basal medium, adherent plates; RSU: Rh30 cells, sphere medium, ultra-low attachment plates; RSA: Rh30 cells, sphere medium, adherent plates; RRA: Rh30 cells, RPMI-1640 medium, adherent plates.

Supplementary Figure 2

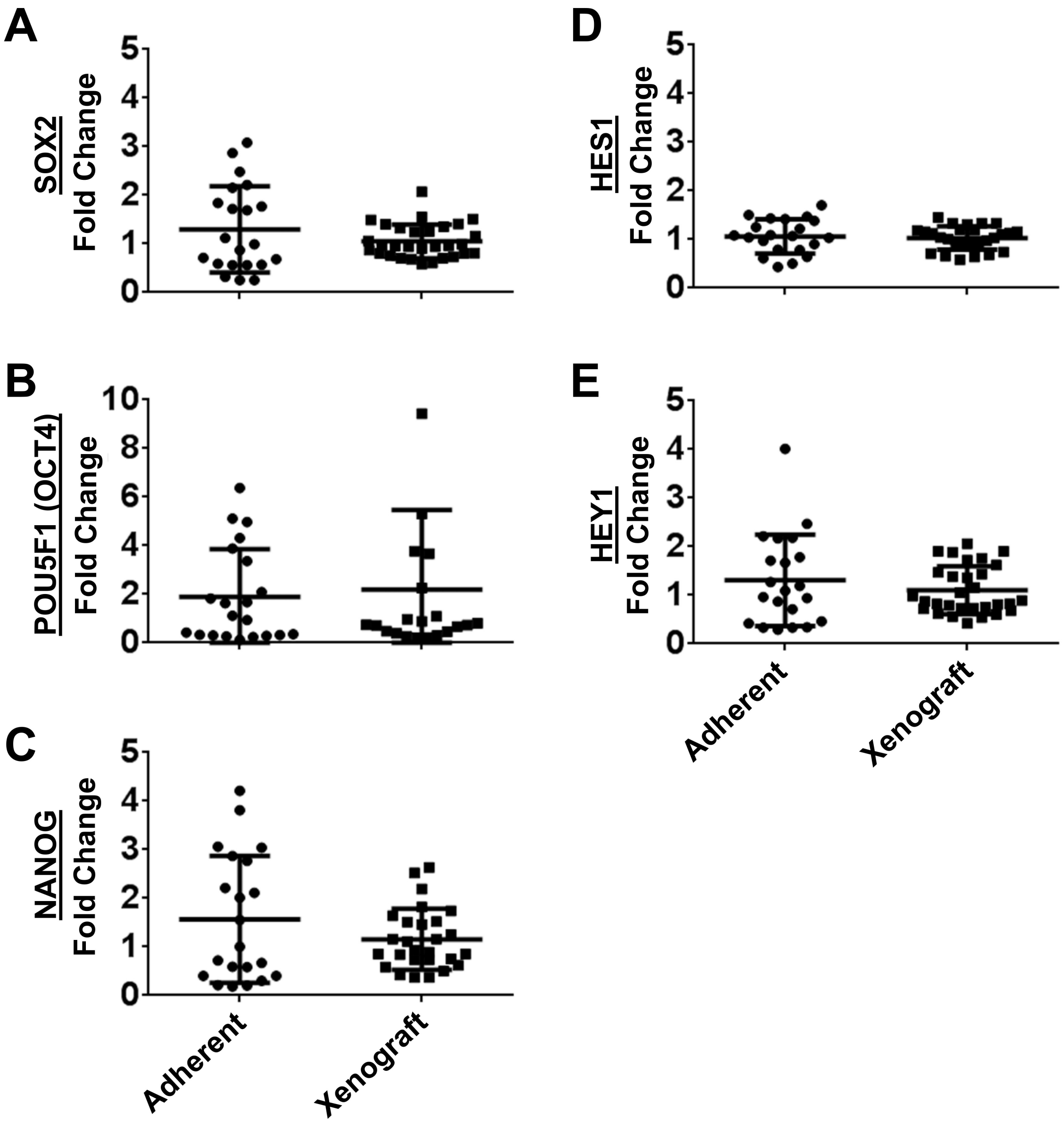

Figure S2. Stem cell and Notch pathway genes are not differentially expressed between tumors resulting from cells grown as adherent cells versus spheres. (A) qRT-PCR for stem cell genes *SOX2, POU5F1,* and *NANOG*, respectively, in adherent and sphere tumors. **(B)** qRT-PCR for Notch pathway genes *HES1* and *HEY1*, respectively, in adherent and sphere tumors.

Biology Open • Supplementary information

Supplementary Figure 3

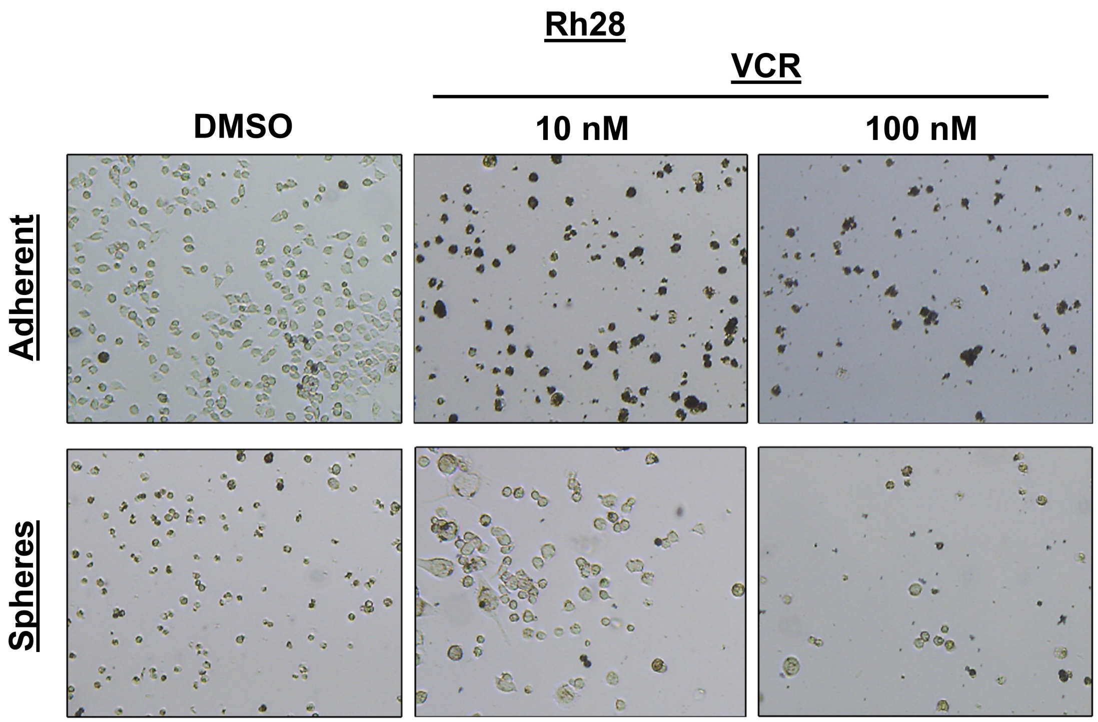

Figure S3. FP-RMS Rh28 spheres exhibit chemoresistance. Representative images of resistance colonies in Rh28 spheres after treatment with high concentrations (10nM and 100nM) of VCR. All the adherent cells died at these concentrations, shown by the dark floating cells.

Biology Open • Supplementary information

Supplementary Table 1

Oligos used for qPCR	
**Oligo**	**Sequence (5'-3')**
GAPDH FW	ATG GGG AAG GTG AAG GTC G
GAPDH REV	GGG GTC ATT GAT GGC AAC AAT A
POU5F1 (OCT4) FW	GGG CTC TCC CAT GCA TTC AAA C
POU5F1 (OCT4) REV	CAC CTT CCC TCC AAC CAG TTG C
NANOG FW	AGT CCC AAA GGC AAA CAA CCC ACT TC
NANOG REV	TGC TGG AGG CTG AGG TAT TTC TGT CTC
SOX2 FW	TAC AGC ATG TCC TAC TCG CAG
SOX2 REV	GAG GAA GAG GTA ACC ACA GGG
HEY1 FW	GTT CGG CTC TAG GTT CCA TGT
HEY1 REV	CGT CGG CGC TTC TCA ATT ATT C
HES1 FW	TCA ACA CGA CAC CGG ATA AAC
HES1 REV	GCC GCG AGC TAT CTT TCT TCA
P75 FW	CAG GCT TTG CAG CAC TCA C
P75 REV	CTG CTG CTG TTG CTG CTT CT
PAX6 FW	TTT GCC CGA GAA AGA CTA GC
PAX6 REV	CAT TTG GCC CTT CGA TTA GA
TUJ1 FW	CGA TGC CAT GCT CAT CAC
TUJ1 REV	CCC AGT ATG AGG GAG ATC GT
ALP L FW	GGG TCA GCT CCA CCA CAA
ALP L REV	GCA TTG GTG TTG TAC GTC TTG
COL1A1 FW	CCC CTG GAA AGA ATG GAG AT
COL1A1 REV	AAT CCT CGA GCA CCC TGA
OC FW	GGC GCT ACC TGT ATC AAT GG
OC REV	TCA GCC AAC TCG TCA CAG TC

Oligos used for semi-quantitative RT-PCR	
**Oligo**	**Sequence (5'-3')**
NOTCH1 FW	GCC GCC TTT GTG CTT CTG TTC
NOTCH1 REV	CCG GTG GTC TGT CTG GTC GTC
NOTCH3 FW	TCT TGC TGC TGG TCA TTC TC
NOTCH3 REV	TGC CTC ATC CTC TTC AGT TG
GAPDH FW	GAG AGA CCC TCA CTG CTG
GAPDH REV	GAT GGT ACA TGA CAA GGT GC

Biology Open • Supplementary information
